# What factors predict drivers’ self-reported lane change violation behavior at urban intersections? A study in China

**DOI:** 10.1371/journal.pone.0216751

**Published:** 2019-05-15

**Authors:** Xiaoxiao Wang, Liangjie Xu, Yanping Hao

**Affiliations:** 1 School of Transportation, Wuhan University of Technology, Wuhan, Hubei, China; 2 School of Civil and Transportation Engineering, Henan University of Urban Construction, Pingdingshan, Henan, China; Middlesex University, UNITED KINGDOM

## Abstract

Lane change violations are a major cause of traffic conflicts and accidents at urban intersections and one of many road-safety issues in China. This study aims to explore the socio-psychological factors underlying drivers’ motivation for lane change violation behavior at urban intersections and examines how these factors predict this violation behavior. A self-reported questionnaire is designed by applying the construct of the theory of planned behavior (TPB) to collect data. Five hundred-six valid responses are received from the questionnaire survey conducted on the Internet in China. The data are then analyzed using structural equation modeling (SEM). The results of the analysis show that behavioral intention is the strongest predictor of self-reported lane change violation behavior at urban intersections. Perceived behavioral control has both direct and indirect effects on self-reported lane change violation behavior. Furthermore, attitude, subjective norms and perceived behavioral control are found to have significant correlations with drivers’ intention of lane change violations at urban intersections. The results of this study could provide a reference for designing more effective interventions to modify drivers’ lane change violation behavior at urban intersections.

## Introduction

Traffic injuries at intersections account for a large proportion of total traffic-related injuries. In the United States in 2012, 2.5 million traffic crashes occurred at intersections, of which 2,850 were fatal [1]. In China, approximately 30% of total road fatalities occur at intersections [2]. The high number of intersection-related crashes can be partially attributed to drivers’ frequent traffic violation behaviors to a large extent [3]. Lane change violations have a great impact on road traffic accidents. According to statistics, during 2010, 5,464 accidents were related to lane change violations in China, with 1,046 deaths and 5,495 injured [4].

As for the legal status of driving behavior at intersections in China, Article 44 and Article 45 of the Road Traffic Safety Law clearly stipulate that drivers shall strictly observe the indications of traffic lights, traffic signs and traffic line markings when passing through intersections [5]. Lane change violations at intersections are a major cause of traffic conflicts, which can develop into road traffic accidents. To make drivers abide by the law, photo-enforcement cameras are installed at many intersections to deter drivers from making traffic violations. Although traffic violations at intersections could be significantly reduced by the presence of cameras [6–7], there is still a tendency among some drivers to cross the solid lane line before the stop line to avoid delays or to keep on the right route at many intersections in China.

Previous studies of lane change violations at urban intersections have mainly focused on detection and discriminant methods [8–9]. However, to the best of our knowledge, few studies have been carried out to provide a thorough investigation of drivers’ motivation for this behavior. Although acknowledging the frequency of lane change violations at urban intersections, existing traffic management measures in some cities in China have been unsuccessful in reducing this behavior. Therefore, it is important to examine the factors underlying this behavior, especially those capable of being modified. The present study aims to identify the socio-psychological factors influencing drivers’ lane change violations at urban intersections based on the theory of planned behavior (TPB) to support further behavioral interventions to reduce this behavior.

The remainder of the paper is organized as follows: First, we present a literature review of TPB, and then, hypotheses and a conceptual model are proposed. Second, we describe the participants and procedure of the survey and the measure constructs and questionnaire items. Third, the data analysis method and model used in our study are presented. Fourth, we present the results of our study. Finally, we provide an in-depth discussion of the results, followed by our conclusions.

### Literature review

The theory of planned behavior (TPB) is a theoretical model to explain the decision process of individual behavior first proposed by Ajzen [10]. According to TPB, attitude (one’s evaluation of a certain behavior either positively or negatively), subjective norms (one’s perspective on a certain behavior under the influence of important judgments by others), and perceived behavioral control (one’s perceived conducting of a certain behavior either easily or with difficulty) together form one’s behavioral intention and behavior [10]. Based on TPB, numerous studies have found that psychological factors are strongly associated with drivers’ traffic violation behaviors.

Atombo et al. conducted a study on how motivational factors influence drivers’ speeding violations. The study showed that all components of TPB had strong significant correlations with drivers’ intention toward speeding violations [11]. Benson et al. investigated the motivations behind texting behavior while driving. Their results showed that moral norm was the strongest predictor of intention toward texting-while-driving violations [12]. Studies by Palat et al. revealed that attitude and descriptive norms accounted for significant parts of the total effects on intention toward yellow-light-running violations [13]. Li et al.’s study showed that perceived behavioral control together with social environment had an indirect influence on competitive driving behavior [14]. Studies by Mohamed et al. demonstrated that attitude toward traffic safety was a significant predictor of drivers’ aggressive driving behavior [15]. Jiang et al.’s study implied that self-reported fatigued driving behavior was significantly influenced by factors of subjective norm, perceived behavioral control and intention [16]. In all cases, the above studies confirmed that illegal driving behaviors had strong significant correlations with TPB factors.

There are no known studies exploring the socio-psychological factors underlying drivers’ decisions of lane change violations at urban intersections based on TPB. However, there are some similarities between drivers’ decisions in lane change violations at urban intersections and other driving violations, such as saving time, pursuing convenience, reaching a destination faster, and so on. Therefore, we attempt to investigate drivers’ motivation for lane change violations at urban intersections based on the TPB.

### Hypotheses and the proposed model

**Fig 1:**
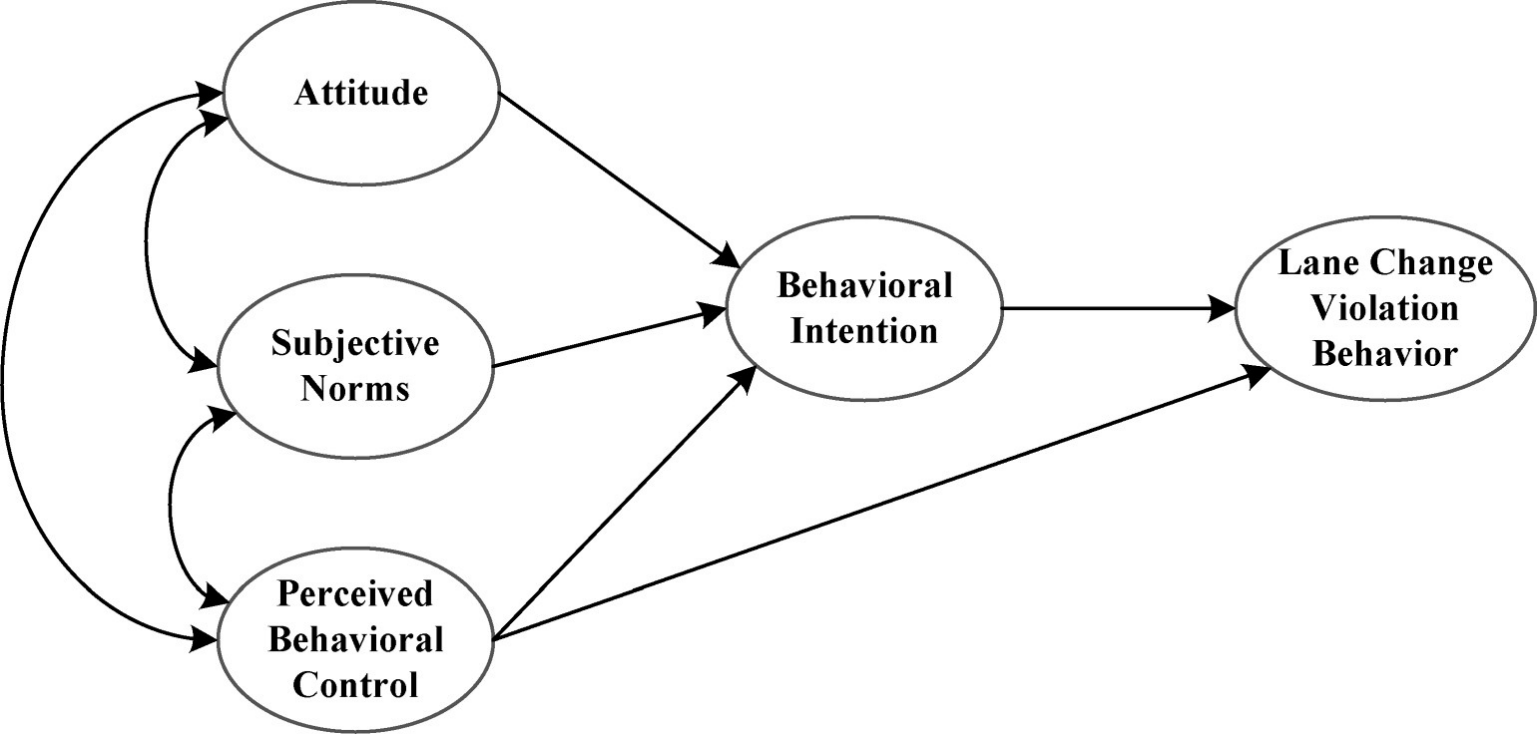
Proposed model. The proposed model describes the hypothesized relationships among the variables. One-way straight arrows represent one-way path relationships, and two-way arrows represent two-way path relationships between variables.

The factors underlying the motivation for lane change violations at intersections are not clear. Yet it is evident from the review that TPB constructs relate significantly to drivers’ intention [11–16] and driving violation behavior is found to have a significant correlation with drivers’ intention [12, 14–16] as well as perceived behavioral control [14, 16]. Therefore, based on the TPB and the review of related studies, we formulated some hypotheses and developed a conceptual model. The hypotheses are shown in Table 1 and the proposed model is shown in Fig 1.

**Table 1:** Hypotheses of this study.

Hypotheses	Description
**H1**	There is a significant correlation among the attitude toward lane change violations at urban intersections, subjective norms and perceived behavioral control.
**H2**	The behavioral intention toward lane change violations at urban intersections is predicted by attitude, subjective norms and perceived behavioral control.
**H3**	Self-reported lane change violation behavior at urban intersections is predicted by behavioral intention toward lane change violations.
**H4**	Self-reported lane change violation behavior at urban intersections is predicted by perceived behavioral control over lane change violations.
**H4a**	There is an association between perceived behavioral control and self-reported lane change violation behavior at urban intersections through behavioral intention.

## Survey and characteristics of items

### Ethics statement

This study was approved by the Institutional Review Committee of the School of Transportation at Wuhan University of Technology. Before participants began the online survey, they were shown a text on the purpose and use of the survey and voluntarily decided whether to continue filling out the questionnaire. Participants were allowed to terminate the online survey at any time during the investigation process according to their own circumstances. All information related to the participants is strictly confidential. As lane change violation driving is both illegal and risky, participants were reminded of the danger of this behavior at the end of the questionnaire.

### Participants and survey procedure

In our study, questionnaire survey was conducted to collect data. There were three parts in the questionnaire. The first part was an introduction instructing participants how to complete the questionnaire. The second part collected participants’ demographic information, such as sex, age, driving experience, education, and driving frequency. The third part was designed to measure the TPB factors, lane change violation intentions and self-reported behaviors of participants.

Before the formal survey, a pretest with a small group (50 people) was carried out to exclude those unreliable and invalid items using a correlation and consistency test [16]. The retained items of every construct will be described in the next section. The formal questionnaire survey lasted for two months and was conducted online with the help of Changsha Questionnaire Star Network Technology Company Limited, which has a professional online platform providing questionnaire and assessment services in China. It should be noted that only participants 18 and above and with a driving license were invited. Moreover, participants were not allowed to take part in the survey more than once when they had the same Internet protocol address.

In total, of 621 questionnaires distributed, 506 were found valid, representing 81.5% of the total questionnaires administered. Participants’ demographic information is described in Table 2. Participants included 280 (55.3%) male and 226 (44.7%) female drivers. Descriptive statistics showed that most of the respondents (35.8%) were between the ages of 30 and 39. 36.4% of respondents had an undergraduate degree. Furthermore, 34.6% had driving experiences of 6 to 10 years, and 32.0% drove their cars from 6 to10 hours per week. For further details, see Table 2.

**Table 2:** Summary of respondents’ demographic information (*N* = 506).

Items	Freq.	Percent (%)	Items	Freq.	Percent (%)
**Gender**			**Age**		
Male	280	55.3	18–29	113	22.3
Female	226	44.7	30–39	181	35.8
**Education**			40–49	127	25.1
Below senior high school	92	18.2	≥ 50	85	16.8
Senior high school	151	29.8	**Driving frequency (hours per week)**		
Undergraduate	184	36.4	0–5	99	19.6
Above undergraduate	79	15.6	6–10	162	32.0
**Driving experience**			11–15	111	21.9
< 2 years	130	25.7	16–20	71	14.0
2–5 years	170	33.6	> 20	63	12.5
6–10 years	175	34.6			
> 10 years	31	6.1			

### Constructs and corresponding items' variables

The constructs and corresponding items were adopted based on the proposed model and related studies on traffic violations [10–16]. There were five constructs and 15 items in the third part of our questionnaire, which are shown in Table 3.

**Table 3:** Statements of constructs and corresponding items.

Constructs	Items	Statements
**Behavioral intention (BI)**	BI1	It is likely that I intend to change lanes by crossing the solid lane line at urban intersections if I feel my car is capable of doing so in any driving condition.
	BI2	It is likely that I intend to change lanes by crossing the solid lane line at urban intersections if my car is in the wrong lane.
	BI3	It is likely that I intend to change lanes by crossing the solid lane line at urban intersections if the queue in front of my lane is longer than in the other lane.
**Attitude (AT)**	AT1	It is convenient and saves times when I pass urban intersections by making a lane change across the solid lane line.
	AT2	Lane changes by crossing the solid lane line at urban intersections enable me to arrive at my destination more quickly.
	AT3	Lane changes by crossing the solid lane line at urban intersections would not affect traffic.
	AT4	Lane changes by crossing the solid lane line at urban intersections give me a sense of accomplishment.
**Subjective norms (SN)**	SN1	My family wouldn’t stop me from making lane change violations at urban intersections.
	SN2	My friends wouldn’t stop me from making lane change violations at urban intersections.
	SN3	The police wouldn’t ticket drivers for making lane change violations at urban intersections [16].
**Perceived behavioral control (PBC)**	PBC1	I am capable of evaluating all situations carefully enough when I change lanes at urban intersections.
	PBC2	When I change lanes at urban intersections, my capability can match the high challenge of the situations on the road.
	PBC3	Obeying the lane markings at urban intersections depends on the circumstances, not on me.
**Self-reported lane change violation behavior at intersections (LCV)**	LCV1	How many times have you crossed the solid lane line at urban intersections in the past two years?
	LCV2	How many times have you been punished for lane change violations at urban intersections in the past two years?

The measure of a driver’s behavioral intention (BI) to cross the solid lane line at urban intersections was obtained from three items. Participants rated the extent to which they would feel like performing a lane change violation in three described scenarios. The BI construct was measured on a 5-point scale, ranging from 1 = ‘‘strongly disagree” to 5 = ‘‘strongly agree”. Cronbach’s alpha (*α*) was 0.903.

Four items were used to measure a driver’s attitude (AT) toward lane change violations at intersections. The four statements were about attitude of convenience and time-saving, arriving at a destination more quickly, traffic order, and a sense of accomplishment. The participants were asked to select the answer that reflects his/her level of agreement to the statements. The AT construct was measured on a 5-point scale, ranging from 1 = ‘‘strongly disagree” to 5 = ‘‘strongly agree”. Cronbach’s alpha (*α*) was 0.795.

Three items were used to measure subjective norms (SN). Participants rated the extent to which their family members and important friends supported their conducting lane change violations at urban intersections. In addition, participants rated the extent to which the police would ticket them for this behavior [16]. The SN construct was measured on a 5-point scale, ranging from 1 = ‘‘strongly disagree” to 5 = ‘‘strongly agree”. Cronbach’s alpha (*α*) was 0.846.

Perceived behavioral control (PBC) was measured using three items. Participants rated the extent to which they thought conducting a lane change violation at urban intersections was easy, the extent to which they thought it was possible, and the extent to which it depended on their own decision. The SN construct was measured on a 5-point scale, ranging from 1 = ‘‘strongly disagree” to 5 = ‘‘strongly agree”. Cronbach’s alpha (*α*) was 0.788.

Self-reported lane change violation behavior at intersections (LCV) was measured using two items. Participants rated the frequency with which they have conducted lane change violation behavior at urban intersections, and at which they have been punished for this behavior. The LCV construct was measured on a 5-point Likert scales, ranging from never (1) to very often (5). Cronbach’s alpha (*α*) was 0.867.

## Data analysis and model development

Descriptive statistics were conducted to reveal the profiles of all the items and the five constructs. A Pearson’s chi-square test was used to examine the gender and age differences of drivers’ self-reported lane change violation behavior. Pearson correlation analysis was performed to explore the relations among all the items. All of the above data analyses were conducted using the IBM SPSS v 25.0 program.

Explanatory factor analysis (EFA) was utilized to guide the intrinsic structure of the large set of item variables [17]. A Kaiser-Meyer-Olkin (KMO) test was used to verify the adequacy of the sample, and the KMO value should above 0.5 [17–18]. Bartlett’s test of sphericity was used to verify whether the data were appropriate for factor analysis [17–18]. Principle component factor analysis with varimax-rotation was performed for extraction factor and factor rotation in EFA, which was conducted using the IBM SPSS v 25.0 program.

To test the theoretical model proposed in this research, we used structural equation modeling (SEM), which is an effective technique to examine hypotheses about relationships among observed and latent variables [15, 19–21]. A SEM model comprises two parts: the measurement model and structural model [15, 19–22]. The measurement model describes the link between observed variables and latent variables, and the structural model uses the simultaneous equations to relate latent variables to each other [15, 19–22].

The measurement model was tested using confirmatory factor analysis (CFA) [17–23]. In CFA, the reliability, convergent validity, and discriminant validity of the constructs were tested. The structural model was tested to make a path analysis of hypothesized relationships between constructs.

In our study, both CFA and the structural model test were performed using the AMOS 24.0 software package. The estimation methods available in AMOS include maximum likelihood (ML) estimation, generalized least squares (GLS), unweight least squares (ULS), scale-free least squares (AFLS), and asymptotically distribution-free (ADF) [21–22]. ML is the default method in AMOS and is the one most commonly used in a SEM test [15, 21–23]. When the data are approximately normal [21–23] and the data sample size is medium or large (greater than 500) [21], the ML estimation method is recommended.

Data normality assessment was taken first, prior to estimating the measurement model and the structural model. As Kline suggested that tests such as z-test might not be helpful in some situations, we tested normality based on absolute values of skewness and kurtosis [23]. For acceptation of normality, Kline suggested that absolute values of skewness and kurtosis should be less than 3.0 and 8.0 [23], respectively, while Singh et al. suggested that skewness and kurtosis values should both range between -2.0 and 2.0 [17]. We adopted the suggestion of Singh et al. as our criterion.

The CFA model and the structural model testing fits were verified using several fit indices, such as chi-square divided by degrees of freedom (*χ*^2^/DF), the root mean square error of approximation (RMSEA), comparative fit index (CFI) and so on [21–27].

## Results

### Descriptive statistics

**Fig 2:**
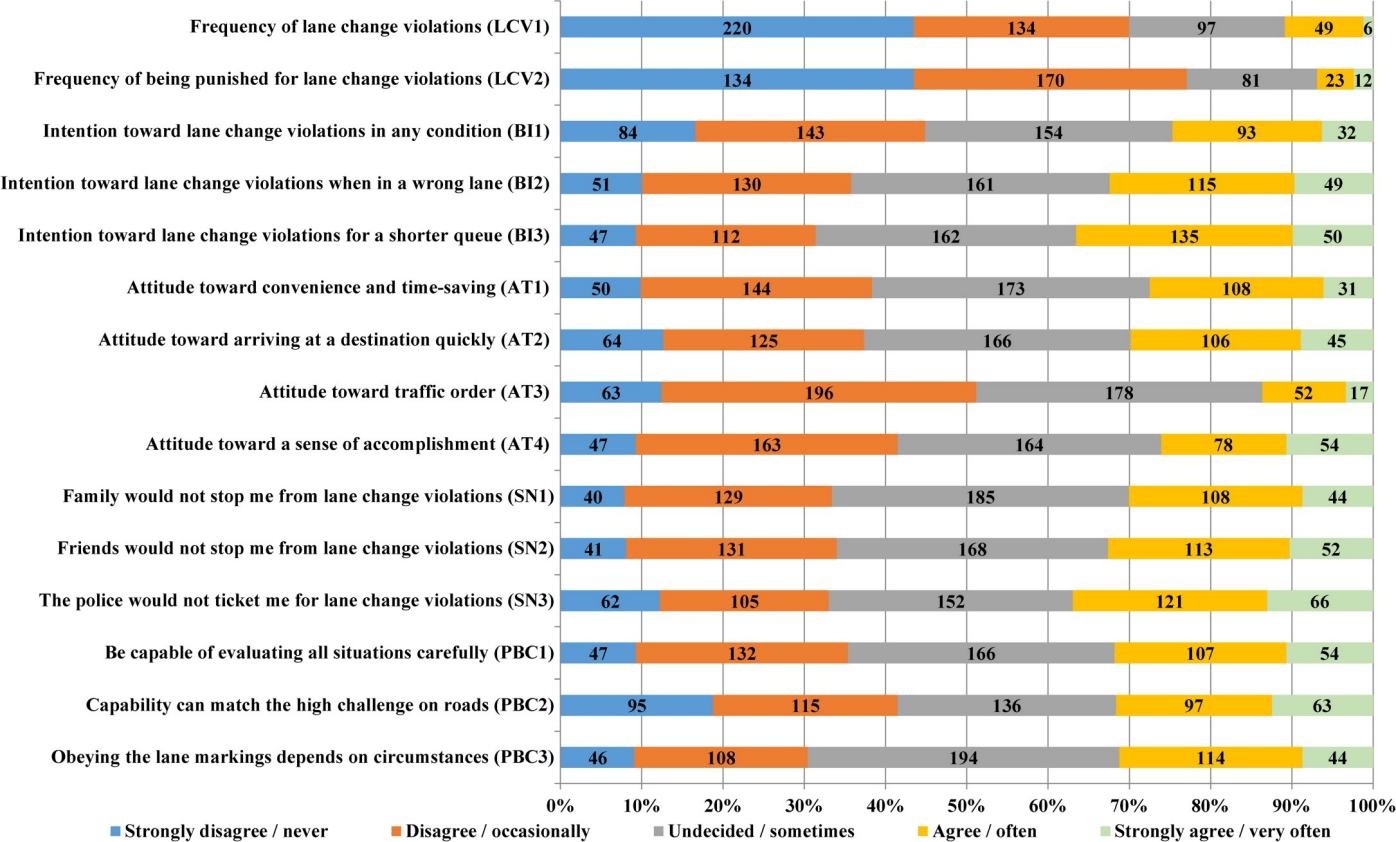
Frequencies of the responses to each question (*N* = 506).

Approximately three fifths (64.6%) of participants stated they had experienced lane change violations at intersections and 48.4% reported they had been punished for this violation. Fig 2 displays the details of responses to each question of our questionnaire.

The score values of the five constructs were calculated using the average score values of their corresponding items. Means and standard deviations of constructs and items are reported in Table 4 as well as skewness and kurtosis values of all items.

**Table 4:** Descriptive statistics of the constructs and items (*N* = 506).

Constructs		Items	M	SD	Skewness	Kurtosis
**BI**	M_BI_ = 2.91 SD_BI_ = 1.03	BI1	2.70	1.137	0.195	-0.741
		BI2	2.96	1.130	0.057	-0.746
		BI3	3.06	1.120	-0.088	-0.724
**AT**	M_AT_ = 2.78 SD_AT_ = 0.84	AT1	2.85	1.058	0.103	-0.600
		AT2	2.89	1.144	0.070	-0.736
		AT3	2.53	0.952	0.415	0.010
		AT4	2.86	1.121	0.338	-0.589
**SN**	M_SN_ = 3.01 SD_SN_ = 0.99	SN1	2.97	1.065	0.091	-0.549
		SN2	3.01	1.104	0.082	-0.688
		SN3	3.05	1.208	-0.064	-0.877
**PBC**	M_PBC_ = 2.94 SD_PBC_ = 0.98	PBC1	2.98	1.128	0.101	-0.718
		PBC2	2.84	1.283	0.124	-1.023
		PBC3	3.00	1.074	-0.027	-0.510
**LCV**	M_LCV_ = 1.91 SD_LCV_ = 0.92	LCV1	2.11	1.051	0.637	-0.508
		LCV2	1.72	0.892	1.131	0.684

The mean of behavioral intention (M_BI_ = 2.91, SD_BI_ = 1.03) is slightly below 3.00 (the midpoint scale in this study). Participants have weak intentions toward lane change violations on average (M = 2.70, SD = 1.137, item with the lowest score) and their intentions are neutral when they have the chance to change to a shorter queue lane (M = 3.06, SD = 1.120, item with the highest score).

The low mean score of attitude (M_AT_ = 2.78, SD_AT_ = 0.84) reveals that many participants have negative attitudes toward lane change violation behavior regarding traffic order (M = 2.53, SD = 0.952, item with the lowest score) and arriving at a destination quickly (M = 2.89, SD = 1.144, item with the highest score).

The mean of subjective norms (M_SN_ = 3.01, SD_SN_ = 0.99) is approximately equal to 3.00, indicating that participants perceive social pressure neutrally regarding that violation in terms of family (M = 2.97, SD = 1.065, item with the lowest score), friends or police (M = 3.05, SD = 1.208, item with the highest score).

The mean of PBC (M_PBC_ = 2.94, SD_PBC_ = 0.98) is slightly below 3.00. The participants believed that it was difficult to conduct lane change violations (M = 2.84, SD = 1.283, item with the lowest score) and that they conducted this behavior neutrally depended on circumstances (M = 3.00, SD = 1.074, item with the highest score).

The low score of self-reported lane change violation behavior (M_LCV_ = 1.91, SD_LCV_ = 0.92) shows that participants have a low frequency of this behavior (M = 2.11, SD = 1.051) or are less likely to be punished for this violation (M = 1.72, SD = 0.892).

Table 5 shows the gender and age differences of drivers’ self-reported lane change violation behavior. Pearson’s chi-square test results show that gender (*p* ≤ 0.001) and age (*p* ≤ 0.001) have significance associations with self-reported lane change violation behavior at intersections. Male drivers (M_1_ = 2.41, SD_1_ = 0.969; M_2_ = 1.93, SD_2_ = 0.903) are more likely to engage in this violation than female drivers (M_1_ = 1.73, SD_1_ = 1.026; M_2_ = 1.47, SD_2_ = 0.812). Those drivers aged 18–29 (M_1_ = 2.38, SD_1_ = 0.929; M_2_ = 1.98, SD_2_ = 0.732) are more likely to engage in this violation than the other age groups.

**Table 5:** Self-reported lane change violation behavior by gender and age.

Items		LCV1				LCV2			
		(1)	(2)-(5)	M_1_	SD_1_	(1)	(2)-(5)	M_2_	SD_2_
**Gender**	Male	9.5%	45.8%	2.41	0.969	21.0%	34.4%	1.93	0.903
	Female	25.9%	18.8%	1.73	1.026	30.6%	14.0%	1.47	0.812
	$\chi_{1}^{2}$	93.81^***^				49.31^***^			
**Age**	18–29	4.4%	18.0%	2.38	0.929	5.9%	16.4%	1.98	0.732
	30–39	14.8%	20.9%	2.14	1.187	20.8%	15.0%	1.75	1.034
	40–49	7.7%	17.4%	2.05	0.916	12.9%	12.2%	1.65	0.801
	≥ 50	8.5%	8.3%	1.76	0.996	12.1%	4.7%	1.42	0.792
	$\chi_{2}^{2}$	55.76^***^				73.46^***^			

*Notes*: (1) never, (2) occasionally, (3) sometimes, (4) often, (5) very often.

^***^ *p* ≤ 0.001.

Table 6 presents Pearson correlations among all items. The results show that all items are significantly positively associated with self-reported lane change violation behavior at urban intersections.

**Table 6:** Pearson correlations among items.

Items	BI			AT				SN			PBC			LCV	
	BI1	BI2	BI3	AT1	AT2	AT3	AT4	SN1	SN2	SN3	PBC1	PBC2	PBC3	LCV1	LCV2
**BI1**	1	.768^**^	.721^**^	.379^**^	.313^**^	.364^**^	.331^**^	.432^**^	.437^**^	.391^**^	.390^**^	.477^**^	.371^**^	.332^**^	.324^**^
**BI2**		1	.780^**^	.386^**^	.305^**^	.392^**^	.349^**^	.445^**^	.448^**^	.456^**^	.459^**^	.415^**^	.397^**^	.379^**^	.395^**^
**BI3**			1	.361^**^	.262^**^	.313^**^	.349^**^	.445^**^	.448^**^	.420^**^	.399^**^	.381^**^	.365^**^	.313^**^	.337^**^
**AT1**				1	.304^**^	.559^**^	.490^**^	.357^**^	.347^**^	.287^**^	.274^**^	.263^**^	.271^**^	.110^*^	.152^**^
**AT2**					1	.579^**^	.550^**^	.255^**^	.206^**^	.324^**^	.207^**^	.164^**^	.171^**^	.104^*^	.154^**^
**AT3**						1	.506^**^	.314^**^	.267^**^	.319^**^	.232^**^	.228^**^	.230^**^	.141^**^	.131^**^
**AT4**							1	.425^**^	.383^**^	.487^**^	.206^**^	.193^**^	.139^**^	.139^**^	.194^**^
**SN1**								1	.702^**^	.641^**^	.370^**^	.328^**^	.286^**^	.243^**^	.245^**^
**SN2**									1	.611^**^	.393^**^	.334^**^	.316^**^	.238^**^	.298^**^
**SN3**										1	.357^**^	.254^**^	.276^**^	.264^**^	.312^**^
**PBC1**											1	.664^**^	.648^**^	.324^**^	.313^**^
**PBC2**												1	.369^**^	.236^**^	.232^**^
**PBC3**													1	.300^**^	.245^**^
**LCV1**														1	.775^**^
**LCV2**															1

*Notes*: ^*^ *p* ≤ 0.05,

^**^ *p* ≤ 0.01,

^***^ *p* ≤ 0.001.

### The results of EFA

Explanatory factor analysis (EFA) was utilized to examine the intrinsic structure of the item set and to extract the principal factors. EFA was developed based on a sample of 253 from original data (*N* = 506). Before EFA, a Kaiser-Meyer-Olkin test and Bartlet’s test were taken. The results showed that KMO = 0.83 > 0.5, which indicates the adequacy of the sample [17–18]. Bartlet’s test of sphericity was significant (*p* ≤ 0.001) with Chi-square = 2175.012, which indicates it was suitable to conduct EFA [17–18]. Principle component factor analysis with varimax-rotation was performed to extract factors from the total 15 items. The results of EFA are shown in Table 7.

Each item was assigned to a factor according to its factor loading. In our study, items with factor loadings above 0.5 and without cross-loadings were retained [16–18]. As shown in Table 7, all factor loadings of items were above the threshold value of 0.5, and no items were eliminated. Using EFA, five factors were extracted from 15 items: LCV, BI, AT, SN and PBC. These factors could explain 76.95% of the total variance.

**Table 7:** Results of EFA (*N* = 253).

Items		Factor loadings	Variance explained (%)	Cumulative variance explained (%)
**Factor: LCV**	LCV1	0.893	12.40%	12.40%
	LCV2	0.894		
**Factor: BI**	BI1	0.852	17.09%	29.49%
	BI2	0.785		
	BI3	0.827		
**Factor: AT**	AT1	0.578	16.10%	45.59%
	AT2	0.791		
	AT3	0.845		
	AT4	0.707		
**Factor: SN**	SN1	0.832	16.58%	62.17%
	SN2	0.808		
	SN3	0.789		
**Factor: PBC**	PBC1	0.846	14.78%	76.95%
	PBC2	0.676		
	PBC3	0.817		

*Notes*: Extraction method: Principal Component Analysis. Rotation method: Varimax.

### The results of CFA

To test the measurement model, confirmatory factor analysis (CFA) was conducted after EFA based on a sample of 253. The data normality assessment results are shown in Table 8. For all items, skewness values range between -0.096 and 1.051, and kurtosis values range between -0.967 and 0.571; both the absolute values of kurtosis and skewness are less than 2.0. Thus, the normality of the items variables is acceptable and maximum likelihood (ML) estimation could be used for CFA.

**Table 8:** Normality assessment of the sample for CFA (*N* = 253).

Variables	Min	Max	M	SD	Skew	Kurtosis
**BI1**	1	5	2.72	1.154	0.214	-0.815
**BI2**	1	5	2.98	1.123	0.013	-0.754
**BI3**	1	5	3.08	1.101	-0.078	-0.669
**AT1**	1	5	2.89	1.038	0.017	-0.631
**AT2**	1	5	2.91	1.151	0.108	-0.678
**AT3**	1	5	2.51	0.962	0.470	0.060
**AT4**	1	5	2.90	1.101	0.385	-0.563
**SN1**	1	5	2.92	1.088	0.102	-0.604
**SN3**	1	5	2.98	1.139	0.120	-0.762
**SN2**	1	5	3.02	1.247	-0.005	-0.967
**PBC1**	1	5	2.95	1.169	0.070	-0.769
**PBC2**	1	5	2.80	1.263	0.187	-0.955
**PBC3**	1	5	2.97	1.109	-0.096	-0.572
**LCV1**	1	5	2.12	1.021	0.595	-0.470
**LCV2**	1	5	1.77	0.906	1.051	0.571

In CFA, the model fit, construct reliability, convergent validity, and discriminant validity were tested. Fit indices statistics in CFA and recommended criteria are shown in Table 9. The model fit indices satisfy all acceptable criteria, indicating that the model fit the data well.

**Table 9:** Fit indices statistics in CFA (*N* = 253).

Indices	Abbreviation	Observed values	Recommended criteria [21–25]
**Normed chi-square**	*χ*^2^/DF	2.006	1 < *χ*^2^/DF < 3
**Goodness-of-fit index**	GFI	0.922	> 0.90
**Adjusted GFI**	AGFI	0.879	> 0.80
**Root mean square error of approximation**	RMSEA	0.063	< 0.05 good fit < 0.08 acceptable fit
**P value for RMSEA**	PCLOSE	0.065	Non-significant
**Normed fit index**	NFI	0.930	> 0.90
**Comparative fit index**	CFI	0.963	> 0.95
**Parsimony goodness-of-fit index**	PGFI	0.599	> 0.50
**Parsimony-adjusted NFI**	PNFI	0.691	> 0.50

The estimation results of the measurement model are shown in Table 10. The standardized regression weights (Std.R.W.) of the observed variables range from 0.699 to 0.903 (*p* ≤ 0.001), indicating that their respective latent constructs significantly represent the observed variables.

**Table 10:** Estimation results of CFA (*N* = 253).

Constructs	Items	R.W.	Std. R.W.	S.E.	*P* value
**LCV**	LCV1	1.000	0.903		
	LCV2	0.882	0.898	0.075	-^***^
**BI**	BI1	1.000	0.861		
	BI2	1.002	0.887	0.055	-^***^
	BI3	0.959	0.865	0.055	-^***^
**AT**	AT1	1.000	0.715		
	AT2	1.084	0.699	0.137	-^***^
	AT3	0.980	0.756	0.098	-^***^
	AT4	1.089	0.734	0.111	-^***^
**SN**	SN1	1.000	0.817		
	SN2	1.077	0.841	0.077	-^***^
	SN3	1.113	0.793	0.084	-^***^
**PBC**	PBC3	1.000	0.732		
	PBC2	1.266	0.814	0.127	-^***^
	PBC1	1.267	0.880	0.115	-^***^

*Notes*: Regression Weight (R.W.), Standardized Regression Weight (Std. R.W.), Standard Error (S.E.).

^***^ *p* ≤ 0.001.

The convergent validity, discriminant validity and construct reliability were tested to ensure the validity of the survey and the proposed model. All of the results are shown in Table 11. Average variance extracted (AVE) was estimated for testing convergent validity. The results show that all AVE values ranging from 0.528 to 0.811 are above the threshold value of 0.5 and are also below composite reliability (CR) values, indicating sufficient convergent validity [17–18, 21–24]. Discriminant validity is also satisfied since the mean shared variance values (MSVs) < AVEs, average shared variance values (ASVs) < AVEs and also the square root of AVEs (on the diagonal in the Table 11) are greater than all inter-construct correlations [17–18, 22–24]. Finally, the result shows that construct reliability is satisfied since all composite reliability (CR) values are above the threshold of 0.70 [17–18, 22–24]. Overall, all five factors in the measurement model have no convergent and discriminant validity and reliability issues.

**Table 11:** Construct reliability, convergent validity, and discriminant validity.

	CR	AVE	MSV	ASV	LCV	BI	AT	SN	PBC
**LCV**	0.896	0.811	0.218	0.151	0.901 ^a^				
**BI**	0.904	0.759	0.383	0.304	0.467 ^b^	0.871 ^a^			
**AT**	0.817	0.528	0.269	0.176	0.192 ^b^	0.517 ^b^	0.727 ^a^		
**SN**	0.858	0.668	0.383	0.266	0.392 ^b^	0.619 ^b^	0.519 ^b^	0.817 ^a^	
**PBC**	0.851	0.658	0.348	0.233	0.440 ^b^	0.590 ^b^	0.362 ^b^	0.509 ^b^	0.811 ^a^

*Notes*: Composite Reliability (CR), Average Variance Extracted (AVE), Maximum Shared Variance (MSV), and Average Shared Variance (ASV).

^a^ Square root of AVEs.

^b^ Inter-construct correlations.

### Results of the structural model

Path analysis was performed to test the proposed model and investigate the relationships between latent variables based on the total sample (*N* = 506). Kurtosis and skewness values of all the items are shown in Table 4. The absolute values of kurtosis and skewness are less than 2.0; the normality is acceptable and maximum likelihood (ML) estimation could be used for the structural model test.

**Fig 3:**
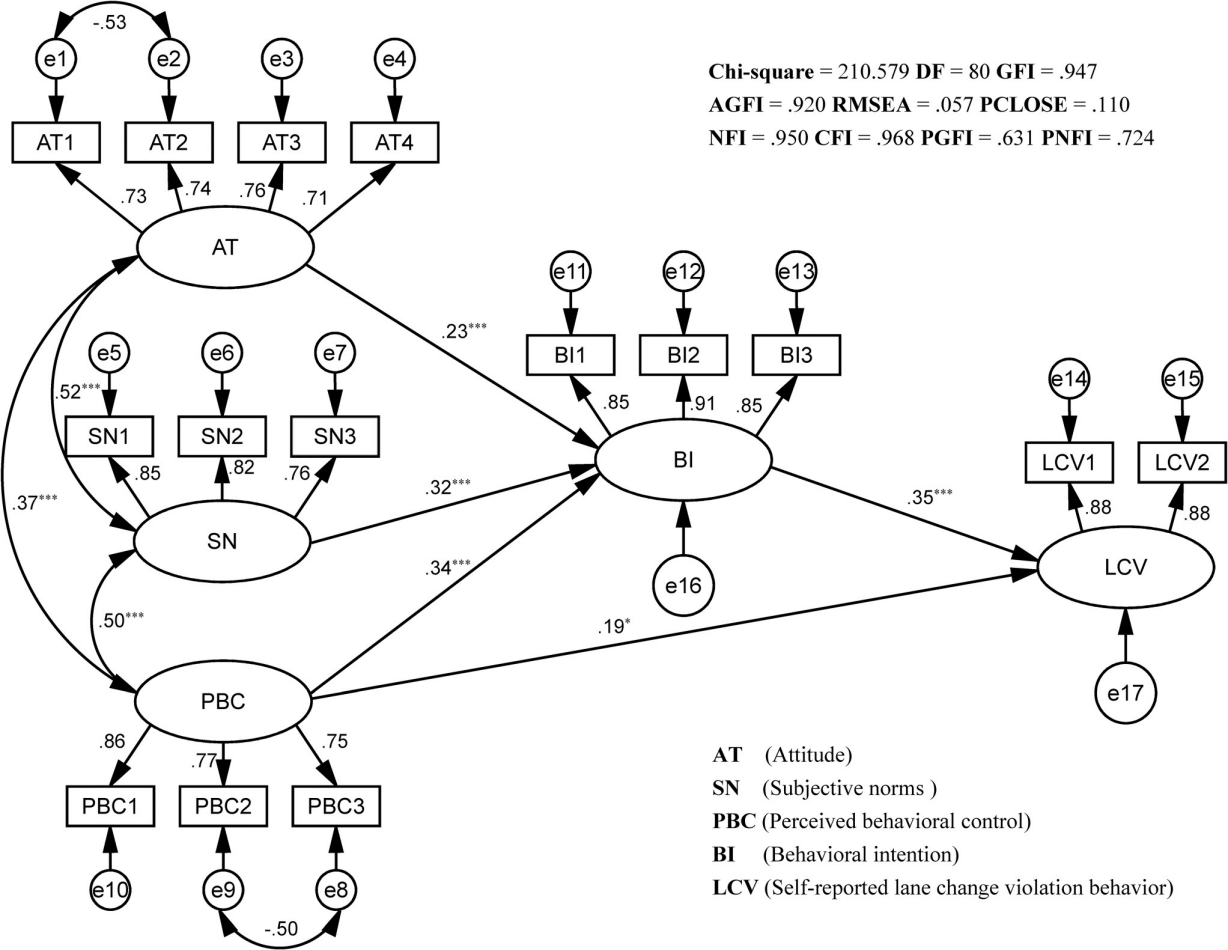
Structural model test results. All paths represent significant standardized regression weights (Std.R.W.). The structural model fit indices shown at the top satisfy all acceptable criteria, indicating an optimal goodness-of-fit path relationship of the structural model. ^*^ *p* ≤ 0.05, ^***^ *p* ≤ 0.001.

Fit indices values of the structural model are shown in Fig 3. The structural model fit indices (*χ*^2^/DF, NFI, CFI, GFI, AGFI, PGFI, PNFI, RMSEA, and PCLOSE) satisfy all acceptable criteria listed in Table 9, indicating a sufficient basis for path analysis. All hypotheses proposed in this study were tested and the path coefficients in the SEM are shown in Fig 3.

The path coefficient between AT and SN is 0.52 (*p* < 0.001), between AT and PBC is 0.37 (*p* < 0.001), and between SN and PBC is 0.50 (*p* < 0.001). All path coefficients among the above constructs are found to be positive, which means significant positive correlations between attitude, subjective norms and PBC. Therefore, hypothesis H1 is supported.

Behavioral intention toward lane change violations at urban intersections is directly predicted by AT (Std.R.W. = 0.23, *p* < 0.001), SN (Std.R.W. = 0.32, *p* < 0.001), and PBC (Std.R.W. = 0.34, *p* < 0.001). Hence, hypothesis H2 is also supported, which means all TBP factors predict behavioral intention toward lane change violations at urban intersections.

Hypothesis H3 states that behavioral intention predicts self-reported lane change violation behavior, which is also supported by the findings in this paper. The effects of BI on LCV (Std.R.W. = 0.35, *p* < 0.001) are positively significant.

The path coefficient from PBC to LCV is significant and positive (Std.R.W. = 0.19, *p* < 0.05). Hypothesis H4 is supported, which means PBC predicts and influences self-reported lane change violation behavior at urban intersections.

To investigate the indirect effect of PBC on LCV via BI, bootstrapping was performed based on 1,000 bootstrap samples. By bootstrapping, a bias-corrected confidence interval (CI) can be obtained [24, 28]. If the confidence interval does not contain zero, the mediation effect is significant [24, 28]. As shown in Table 12, the results indicate that the total effect (0.306), indirect effect (0.118) and direct effect (0.188) of PBC on LCV are all significant, thus, making the case for partial mediation.

**Table 12:** Mediation effect analysis results.

Hypotheses	Path	Std. R.W.	95% Confidence Interval	
			Lower bounds	Upper bounds
**H4a**	PBC → LCV (Total)	0.306	0.200	0.406
	PBC → BI → LCV (Indirect)	0.118	0.074	0.184
	PBC → LCV (Direct)	0.188	0.061	0.304

*Notes*: Standardized Regression Weight (Std.R.W.).

## Discussion

### Gender and age differences in lane change violation behavior

Previous social psychology studies have revealed that males are more risk-seeking than females [29], more competitive than females [30], less altruistic than females [31–32], less cooperative than females [33], more dishonest than females [34], and less harm-averse than females in moral dilemmas [35]. In line with these findings, gender differences were observed in risky driving behavior and traffic violation behavior [16, 36–38].

In our study, a gender difference was also observed in lane change violation behavior at intersections. Males were more likely to engage in lane change violation behavior and to be punished for this behavior than female drivers. Compared with female drivers, male drivers had a stronger intention to perform lane change violations at urban intersections when they had chances to do so. Thus, related traffic management and traffic education should pay more attention to the male driver group.

There were also age differences in drivers’ lane change violation behavior at intersections according to our study. Drivers aged 18–29 were more likely to commit this violation behavior than other age groups. This finding is consistent with previous studies on the driving behavior of young drivers [37–40]. Young drivers are more risky, more confident about their driving skills and had more positive attitudes toward lane change violation behavior. Therefore, young drivers should also be paid more attention to in future interventions for lane change violations at urban intersections.

### Predictors of self-reported lane change violation behavior

Previous studies have found that the TPB could successfully predict and explain speeding [11], texting-while-driving behavior [12], yellow-light-running violation behavior [13], competitive driving behavior [14], aggressive driving behavior [15], and fatigued driving behavior [16]. As expected, the results confirmed that TPB was an effective model in explaining and predicting drivers’ self-reported lane change violation behavior at urban intersections. Specifically, drivers’ lane change violation behavior at urban intersections could be predicted by behavioral intention and PBC. Moreover, attitude, subjective norms and PBC could predict drivers’ behavioral intention toward lane change violations.

In line with previous studies, behavioral intention is the most direct and important predictor of behavior, which further confirms the role of rational decision-making in drivers’ violation behavior [14–16]. As the path analysis results show, behavioral intention has the strongest total effect on self-reported lane change violation behavior at urban intersections, which supports the view that the individual’s intention is a preparation for performing behavior [10].

There is evidence that the direct effects of PBC on both drivers’ behavioral intention and self-reported lane change violation behavior at urban intersections are significant. In addition, PBC has a significant indirect effect on self-reported lane change violations via behavioral intention, indicating that the more confident drivers are about their driving skills, the more willing they are to underestimate traffic conditions and thus to engage in lane change violations at urban intersections.

Furthermore, in our study, attitude is a predictor of drivers’ intention toward lane change violations at urban intersections, suggesting that some drivers are willing to risk crossing the solid lane line at urban intersections when they believe it will bring them meaningful benefits, such as saving time, convenience, or a sense of accomplishment. In line with previous studies [11, 13, 15], this finding demonstrates that intrinsic motivation as an important human factor plays an important role in predicting drivers’ intention toward lane change violations at urban intersections.

Finally, in the present study, subjective norms have significant effects on drivers’ intentions toward lane change violations at urban intersections. This finding agrees with previous results showing that drivers are influenced by family members, friends or the police [11, 16]. This finding also demonstrates that the opinions of important people who disapprove of uncharacteristic behavior may impede their intention toward lane change violations at urban intersections.

### Implications for safety interventions

The results of this study show that behavioral intention is the strongest predictor of lane change violation behavior at urban intersections and correlates strongly with all three components of TPB, which implies that future interventions for lane change violations at urban intersections should be associated with changing drivers’ behavioral intention. To change drivers’ lane change violation intention and behavior is a difficult but important challenge for road safety administrators. However, drivers’ behavioral intention and behavior could be improved by satisfactory improvements in their attitude, subjective norms, and PBC.

Attitude significantly affects drivers’ intention toward lane change violation behavior at urban intersections. Hence, road traffic safety education programs should make drivers realize that it is not worthwhile saving time or obtaining convenience by conducting lane change violations at intersections compared to drivers’ safety. Related road safety regulations education should also be emphasized, as drivers in China have a greater understanding of the illegality of red-light-running behavior at intersections than lane-change-violation behavior. Moreover, serious punishments and fines for drivers’ lane-change-violation of the traffic laws should be strictly enforced.

Subjective norms affect drivers’ intention toward lane change violation behavior at urban intersections. Since drivers’ behavior might be influenced by their family and friends, road traffic safety interventions should be combined with emotional and factual messages. An effective mean of education is to make drivers who intend to engage in lane change violations realize that their violations are not only harmful to themselves but also to their family and friends. What’s more, road traffic safety education programs are not only for drivers, but should also take their parents and children as target groups for interventions. In this way, drivers might obtain positive influences from their family and good friends.

In this study, PBC turns out to significantly predict drivers’ lane change violation intention and behavior. The more control they perceive, the more likely they are to engage in lane change violations at urban intersections. Many drivers do not perceive crossing the solid lane line at intersections as risky driving behavior because they are confident in their driving skills. Therefore, road traffic safety education programs should make drivers realize the serious consequences of traffic accidents caused by lane change violations at urban intersections. Pictures, videos, or victims’ self-reports of lane-change-violation related accidents could play a major role in safety education. At the same time, enforcement should be enhanced. For example, more dedicated cameras for lane change violations should be installed, and the presence of police should also be increased. Under a law enforcement deterrent environment, drivers might find it more difficult to violate the traffic regulation governing lane changes at intersections.

### Limitations of the present study

This study suffers from some limitations. First, a small number of questionnaire items were retained in our study, and further research should appropriately broaden the range of items in all questionnaire constructs. Second, the survey in this study was conducted online, which may have made it impossible for some groups of drivers who were not proficient at using computers and the Internet to participate. Furthermore, all of the measures of the questionnaire were based on a self-reported methodology. Inaccuracies in some participants’ reports could affect the results to some extent.

Finally, our study examines the factors underlying drivers’ lane change violation behavior at intersections using merely a basic model of TPB. However, some studies on drivers or pedestrians’ behavior based on an extended TPB add other influence factors, such as deterring circumstances [13], perceived risk [13, 25], and conformity tendency [25], which proves to be effective. Thus, it is necessary for further studies to introduce additional factors to achieve a better explanation of drivers’ lane change violation behavior at intersections.

## Conclusions

In conclusion, this study applied the TPB model to predict the influences of several socio-psychological factors within a structural equation model on drivers’ self-reported lane change violation behavior at urban intersections. Overall, we found evidence supporting that behavioral intention and PBC are both significantly related to self-reported lane change violation behavior. Behavioral intention is the most direct and important predictor, which is also significantly related to attitude, subjective norms, and PBC. PBC can predict self-reported lane change violation behavior both directly and indirectly. These findings could provide further implications for interventions to modify drivers’ lane change violation behavior at urban intersections.

## Supporting information

S1 File The raw data of this study. (XLS)

S2 File The questionnaire. (PDF)
