# Supplementary material for: What factors predict drivers’ self-reported lane change violation behavior at urban intersections? A study in China
Source: PLoS One. 2019 May 15;14(5):e0216751. doi: 10.1371/journal.pone.0216751 (PMC6519898; doi:10.1371/journal.pone.0216751)
Supplement: S2 File — (PDF) [file pone.0216751.s002.pdf]

A survey on lane change violation behavior at urban intersections

Dear Sir/Madam,

Hello! Thank you very much for participating in this survey during your busy schedule!

This survey is mainly used for research of driving behavior at intersections. The information you provide will help us improve the traffic environment. The results of the survey, without any identifying information, may be published. This survey will be voluntarily participated. If you feel unwell about any of the questions during the questionnaire filling process, you can terminate it at any time. All information will be kept strictly confidential.

Before starting the questionnaire, please confirm that you are at least 18 years old and have a motor vehicle driving license.

I understand that:

- Participation in this survey means I should complete an on-line survey.
- The results of this survey will be used for research and publication without any identifying information.
- My responses will be recorded.
- The researcher will secure all information relating to my responses.
- I may cease to participate at any time during the on-line survey.

☐ I understand and accept the conditions

☐ I do not accept and the investigation is terminated.

Below are a series of questions and statements. The answers to most questions are in the form of a scale. To answer these questions, you need to select the answer that reflects your level of agreement. Thank you for your cooperation!

---

1. I’m no less than 18 years old. ☐ **Yes** ☐ **NO**

2. I have a motor vehicle driving license. ☐ **Yes** ☐ **NO**

3. I have a driving license, but I don't usually drive a car in fact. ☐ **Yes** ☐ **NO**

4. Gender ☐ Male ☐ Female

5. Age ☐ 18-29 ☐ 30-39 ☐ 40-49 ☐ ⩾50

6. Education ☐ Below senior high school ☐ Senior high school ☐ Undergraduate ☐ Above undergraduate

7. Driving experience（the time from you getting a driving license）

☐ Below 2 years ☐ 2-5 years ☐ 6-10 years ☐ Above 10 years

8. How many hours do you drive on average per week?

☐ 0-5 h ☐ 6-10 h ☐ 11-15 h ☐ 16-20 h ☐ Above 20 h

----

9. It is convenient and saves times when I pass urban intersections by making a lane change across the solid lane line.

☐ Strongly disagree ☐ Disagree ☐ Undecided ☐ Agree ☐ Strongly agree

10. Lane changes by crossing the solid lane line at urban intersections enable me to arrive at my destination more quickly.

☐ Strongly disagree ☐ Disagree ☐ Undecided ☐ Agree ☐ Strongly agree

11. Lane changes by crossing the solid lane line at urban intersections would not affect traffic.

☐ Strongly disagree ☐ Disagree ☐ Undecided ☐ Agree ☐ Strongly agree

12. Lane changes by crossing the solid lane line at urban intersections give me a sense of accomplishment.

☐ Strongly disagree ☐ Disagree ☐ Undecided ☐ Agree ☐ Strongly agree

13. My family wouldn't stop me from making lane change violations at urban intersections.

☐ Strongly disagree ☐ Disagree ☐ Undecided ☐ Agree ☐ Strongly agree

14. My friends wouldn't stop me from making lane change violations at urban intersections.

☐ Strongly disagree ☐ Disagree ☐ Undecided ☐ Agree ☐ Strongly agree

15. The police wouldn't ticket drivers for making lane change violations at urban intersections.

☐ Strongly disagree ☐ Disagree ☐ Undecided ☐ Agree ☐ Strongly agree

16. I am capable of evaluating all situations carefully enough when I change lanes at urban intersections.

☐ Strongly disagree ☐ Disagree ☐ Undecided ☐ Agree ☐ Strongly agree

17. When I change lanes at urban intersections, my capability can match the high challenge of the situations on the road.

☐ Strongly disagree ☐ Disagree ☐ Undecided ☐ Agree ☐ Strongly agree

18. Obeying the lane markings at urban intersections depends on the circumstances, not on me.

☐ Strongly disagree ☐ Disagree ☐ Undecided ☐ Agree ☐ Strongly agree

19. It is likely that I intend to change lanes by crossing the solid lane line at urban intersections if I feel my car is capable of doing so in any driving condition.

☐ Strongly disagree ☐ Disagree ☐ Undecided ☐ Agree ☐ Strongly agree

20. It is likely that I intend to change lanes by crossing the solid lane line at urban

intersections if my car is in the wrong lane.

☐ Strongly disagree ☐ Disagree ☐ Undecided ☐ Agree ☐ Strongly agree

21. It is likely that I intend to change lanes by crossing the solid lane line at urban intersections if the queue in front of my lane is longer than in the other lane.

☐ Strongly disagree ☐ Disagree ☐ Undecided ☐ Agree ☐ Strongly agree

22. How many times have you crossed the solid lane line at urban intersections in the past two years?

☐ Never ☐ Occasionally ☐ Sometimes ☐ Often ☐ Very often

23. How many times have you been punished for lane change violations at urban intersections in the past two years?

☐ Never ☐ Occasionally ☐ Sometimes ☐ Often ☐ Very often

Once again, thank you for your participation! We wish you good health and a happy life!

Reminder: Crossing solid lane line on approach of urban intersection is dangerous. Cherish life, stay away from violations!

城市道路交叉口违章变道行为调查

尊敬的先生/女士：

您好！万分感谢您在百忙之中接受本次问卷调查！

本问卷调查主要用于交叉口驾驶行为研究，您所提供的信息将会帮助我们改善交通出行环境。本问卷调查自愿参加，您在问卷填写过程如对部分问题感觉不适，可随时终止填写。本调查相关结果将会发表，但不会涉及您的任何身份信息。您的所有信息我们将会严格保密。

在问卷填写之前，请您确认已年满 18 周岁，并持有机动车驾照。并确认已经知晓以下信息：

- 本调查需要完成网上问卷。
- 调查结果将会用于学术研究和成果发表。
- 我的网上问卷作答会被记录。
- 调查者会对我问卷作答信息严格保密。
- 我可以在问卷调查过程中随时选择终止。

☐ 我已经知晓，同意作答

☐ 我不同意，作答结束

请您根据实际选择最符合的选项，谢谢您的合作！

1、您是否年满 18 周岁？ ☐ 是 ☐ 否

2、您是否拥有机动车驾照？ ☐ 是 ☐ 否

3、您有驾照，但您平时不驾驶车辆。 ☐ 是 ☐ 否

4、您的性别 ☐男 ☐女

5、您的年龄 ☐18-29 岁 ☐30-39 岁 ☐40-49 岁 ☐50 岁及以上

6、您的学历 ☐初中及以下 ☐高中或中专 ☐大专或本科 ☐研究生及以上

7、您的驾龄（拥有驾照时间） ☐2 年以下 ☐2-5 年 ☐6-10 年 ☐10 年以上

8、您平均每周驾驶大约多少小时？ ☐0-5 ☐6-10 ☐11-15 ☐16-20 ☐20 及以上

9、在交叉口进口实线路段变道会使我更快、更方便地通过路口。

☐ 强烈不同意 ☐ 不同意 ☐ 不确定 ☐ 同意 ☐ 强烈同意

10、在交叉口进口实线路段变道可让我更快地到达目的地。

☐ 强烈不同意 ☐ 不同意 ☐ 不确定 ☐ 同意 ☐ 强烈同意

11、在交叉口进口实线路段变道不会对交通秩序造成影响。

☐ 强烈不同意 ☐ 不同意 ☐ 不确定 ☐ 同意 ☐ 强烈同意

12、在交叉口进口实线路段变道让我获得成功感。

☐ 强烈不同意　☐ 不同意 ☐ 不确定　☐ 同意 ☐ 强烈同意

13、我的家人不会阻止我在交叉口进口实线路段变道。

☐ 强烈不同意　☐ 不同意 ☐ 不确定　☐ 同意 ☐ 强烈同意

14、我的朋友不会阻止我在交叉口进口实线路段变道。

☐ 强烈不同意　☐ 不同意 ☐ 不确定　☐ 同意 ☐ 强烈同意

15、在交叉口进口实线路段变道不会受到交警处罚。

☐ 强烈不同意　☐ 不同意 ☐ 不确定　☐ 同意 ☐ 强烈同意

16、在交叉口进口实线路段变道时，我有能力对各种情况进行仔细评估。

☐ 强烈不同意　☐ 不同意 ☐ 不确定　☐ 同意 ☐ 强烈同意

17、在交叉口进口实线路段变道时，我有能力快速应对所有紧急情况。

☐ 强烈不同意　☐ 不同意 ☐ 不确定　☐ 同意 ☐ 强烈同意

18、对于交叉口处的车道标线，我会根据情况选择性遵守。

☐ 强烈不同意　☐ 不同意 ☐ 不确定　☐ 同意 ☐ 强烈同意

19、在任何驾驶环境下，认为可以在交叉口进口道变道时我会变道。

☐ 强烈不同意　☐ 不同意 ☐ 不确定　☐ 同意 ☐ 强烈同意

20、在交叉口实线路段进入错误方向的车道，我会变道进行更正。

☐ 强烈不同意　☐ 不同意 ☐ 不确定　☐ 同意 ☐ 强烈同意

21、当前方车道排队较长，我会在交叉口实线路段变道至排队较短的车道。

☐ 强烈不同意　☐ 不同意 ☐ 不确定　☐ 同意 ☐ 强烈同意

22、近两年，您在交叉口驾驶车辆时违章变道的次数。

☐ 从不　☐ 偶尔 ☐ 有时　☐ 经常 ☐ 十分经常

23、近两年，您因在交叉口违章变道被处罚的次数。

☐ 从不　☐ 偶尔 ☐ 有时　☐ 经常 ☐ 十分经常

再次感谢您的参与！祝您身体健康，生活幸福！

温馨提示：在交叉口进口实线路段变道易发生交通事故。珍爱生命，远离违章！
